# The influence of foehn winds on the incidence of severe injuries in southern Bavaria – an analysis of the TraumaRegister DGU®

**DOI:** 10.1186/s12891-020-03572-z

**Published:** 2020-08-21

**Authors:** Frederik Greve, Karl-Georg Kanz, Michael Zyskowski, Francesca von Matthey, Peter Biberthaler, Stefan Muthers, Andreas Matzarakis, Rolf Lefering, Stefan Huber-Wagner

**Affiliations:** 1 Department of Trauma Surgery, Klinikum rechts der Isar, Technical University of Munich, Ismaninger Straße 22, 81675 Munich, Germany; 2 Research Centre Human Biometeorology, German Meteorological Service, Stefan-Maier-Straße 4, 79104 Freiburg, Germany; 3 Faculty of Health, IFOM – Institute for Research in Operative Medicine, University Witten/Herdecke, Ostmerheimer Str. 200, 51109 Cologne, Germany; 4 Department of Trauma Surgery, Diakonie-Klinikum Schwäbisch Hall, Diakoniestraße 10, 74523 Schwäbisch Hall, Germany

**Keywords:** Foehn wind, Trauma, Polytrauma, Foehn wind Bavaria, TraumaRegister DGU®

## Abstract

**Background:**

Foehn describes a wind which occurs in areas with close proximity to mountains. The presence of foehn wind is associated with worsening health conditions.

This study analyzes the correlation between a foehn typical circulation and the incidence for suffering a severe trauma.

**Methods:**

This is a retrospective, multicentre observational register study. The years from 2013 to 2016 were analyzed for the presence of foehn winds. A logistic regression analysis with the number of daily admitted trauma patients as the primary target value was performed in dependence of foehn winds.

Southern Bavaria is a typical foehn wind region. Individuals were treated in 37 hospitals of Southern Bavaria which participate in the TraumaRegister DGU**®**, an international register that includes all severe trauma patients, mainly in Germany.

We analyzed patients with an Injury Severity Score (ISS) of at least nine with admission to intensive care units or prior death in the emergency room.

**Results:**

6215 patients were enrolled in this study. A foehn-typical circulation was present on 65 days (4.5%). 301 patients (5%) suffered a trauma with an ISS ≥ 9 on a foehn day. The mean ISS was 20.2 (9–75). On average, 4.3 patients (0–15 patients) were admitted on a daily basis due to a severe trauma.

The multivariate regression analysis revealed a daily increase of 0.87 individuals (*p* = 0.004; 95% CI 0.23–1.47) on foehn days. During spring 1.07 patients (*p =* < 0.001; 95% CI 0.72–1.42), in summer 1.98 patients (*p =* < 0.001; 95% CI 1.63–2.32), in fall 0.63 (*p =* < 0.001; 95% CI 0.28–0.97) and on Saturdays, 0.59 patients (*p =* < 0.001; 95% CI 0.24–0.93) were additionally admitted due to severe trauma.

**Conclusion:**

Foehn winds are significantly associated with severe trauma in trauma centers of the TraumaNetzwerk DGU**®**.

## Background

Foehn describes a very strong, warm and dry wind. According to the World Meteorological Organization (1992), foehn is defined as wind on the lee (downwind) side of a mountain which dries and warms during its down-slope descent [1].

**Fig. 1:**
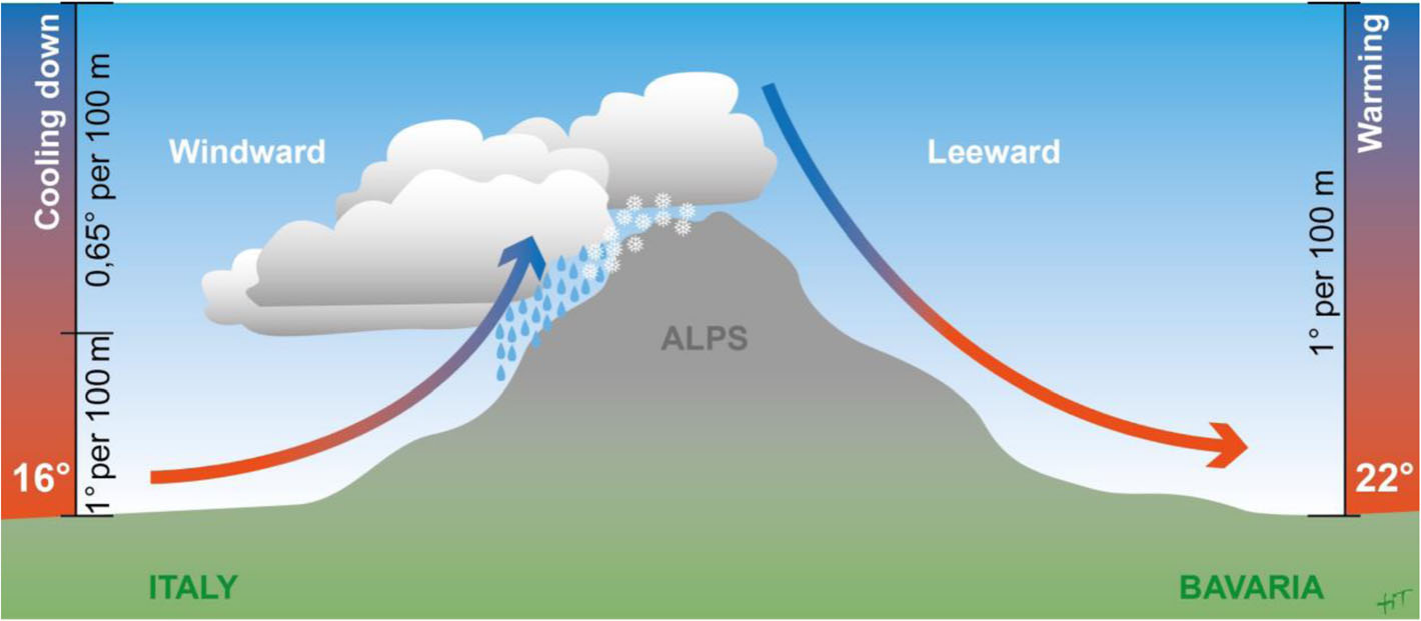
Thermodynamic foehn theory. An air pressure difference with high pressure in the southern region of the Alps causes south to north-directed winds. Dry adiabatic cooling: During its ascent over the mountains, the air cools down proportionally to the decreasing air pressure by 1 °C per 100 m. Cool air saves less water than hot air. This results in the formation of clouds with rain or snow on the southern side of the Alps. Dry adiabatic warming: Due to its higher density, the cool air descends after passing the crest. During its descent, the air warms proportionally by 1 °C per 100 m. This results in a warm dry wind on the northern side of the Alps with a clear vision without clouds

The caused meteorological effects are dry-adiabatically and wet-adiabatically cooling on the upwind side of elevated terrain and dry-adiabatically heating on the downwind side. This results in a warm, dry wind with very good visibility on the downwind side of the mountains [2]. For details on the foehn theory, please see Fig. 1.

The term foehn originated in the Alpine region of Austria, Switzerland and Southern Germany. However, winds that result from the same meteorological phenomena are present all over the world. Examples are Chinook (North America), Helm (United Kingdom), Puelche (Chile), Halny (Poland) or Zonda (Argentina). Bavaria is a typical Southern German foehn region. People often complain about health-related issues worsening due to foehn wind. With time, the expression “foehn illness” developed as a description of the large variety of symptoms patients referred to during days with foehn winds [2].

The first descriptive studies addressing the health aspects of foehn winds go back to the early twentieth century. These studies, also known as the “Innsbruck Foehn Studies”, investigated the effect of foehn winds on the general feeling of students at the University of Innsbruck [3]. In 1933, first results by Rohden et al. described that the individuals of the study population complained about tiredness, headaches, pain and indigestion on foehn days [3]. In the following decades, many diffuse symptoms potentially related to foehn winds were added, including insomnia, nausea, arthritis, general joint pain, wound pain, paraesthesia, weakness, circulation disorders, breathing difficulties, anxiety, nightmares and increased suicides and accidents [4–8]. Apparently, students even tend to postpone examinations and surgeons reschedule surgeries on foehn days [6]. According to Berg et al., the potential influence of foehn winds in the population is equivalent to a “mass psychosis” [9].

Due to the health-related aspects, we hypothesized that the presence of foehn winds might affect the possibility of suffering severe trauma. Potential reasons include higher injury rates due to impacted well-being with influence on driving conditions, sports behavior, or higher suicide rates. It is well-investigated that adverse weather events such as heavy precipitation, frost, heat, and high wind velocity may influence the possibility of accidents, often leading to severe injuries [10–16].

However, the correlation between foehn winds and trauma remains unknown to this day.

Trauma is still the leading cause of death in people younger than 40 years and one of the ten most common causes of death worldwide [17, 18]. Due to the impact of different meteorological conditions, hospitals might adjust resources when an increased patient load is expected.

By use of a large national dataset, this study aims to systematically analyze if an anticipated increase of the incidence of severe trauma on days with foehn winds is justified.

## Methods

### Study design

The study was conducted in a retrospective design. We analyzed patient data derived from the TraumaRegister DGU® (TR-DGU) over a period of 4 years (2013–2016).

### TraumaRegister DGU®

The TraumaRegister DGU® of the German Trauma Society (Deutsche Gesellschaft für Unfallchirurgie, DGU) was founded in 1993. The aim of this multicenter database is a pseudo-anonymized and standardized documentation of severely injured patients.

Data is collected prospectively in four consecutive time phases from the site of the accident until discharge from hospital: A) Pre-hospital phase, B) Emergency room (ER) and initial surgery, C) Intensive care unit (ICU) and D) Discharge. Documentation includes detailed information on demographics, injury pattern, comorbidities, pre- and in-hospital management, course on ICU, relevant laboratory findings including data on transfusion and the outcome of each individual. The inclusion criterion for enrolled patients is admission to hospital via ER with subsequent ICU/Intermediate care unit or reaching the hospital with vital signs and subsequent death before admission to ICU.

The infrastructure for documentation, data management, and data analysis is provided by AUC - Academy for Trauma Surgery (AUC - Akademie der Unfallchirurgie GmbH), a company affiliated to the German Trauma Society. The scientific leadership is provided by the Committee on Emergency Medicine, Intensive Care and Trauma Management (Sektion NIS) of the German Trauma Society. The participating hospitals submit their data in pseudo-anonymized form into a central database via a web-based application. Scientific data analysis is approved according to a peer review procedure established by Sektion NIS.

The present study is in line with the publication guidelines of the TraumaRegister DGU**®** and registered as TR-DGU project ID 2014–024.

### Participants

**Fig. 2:**
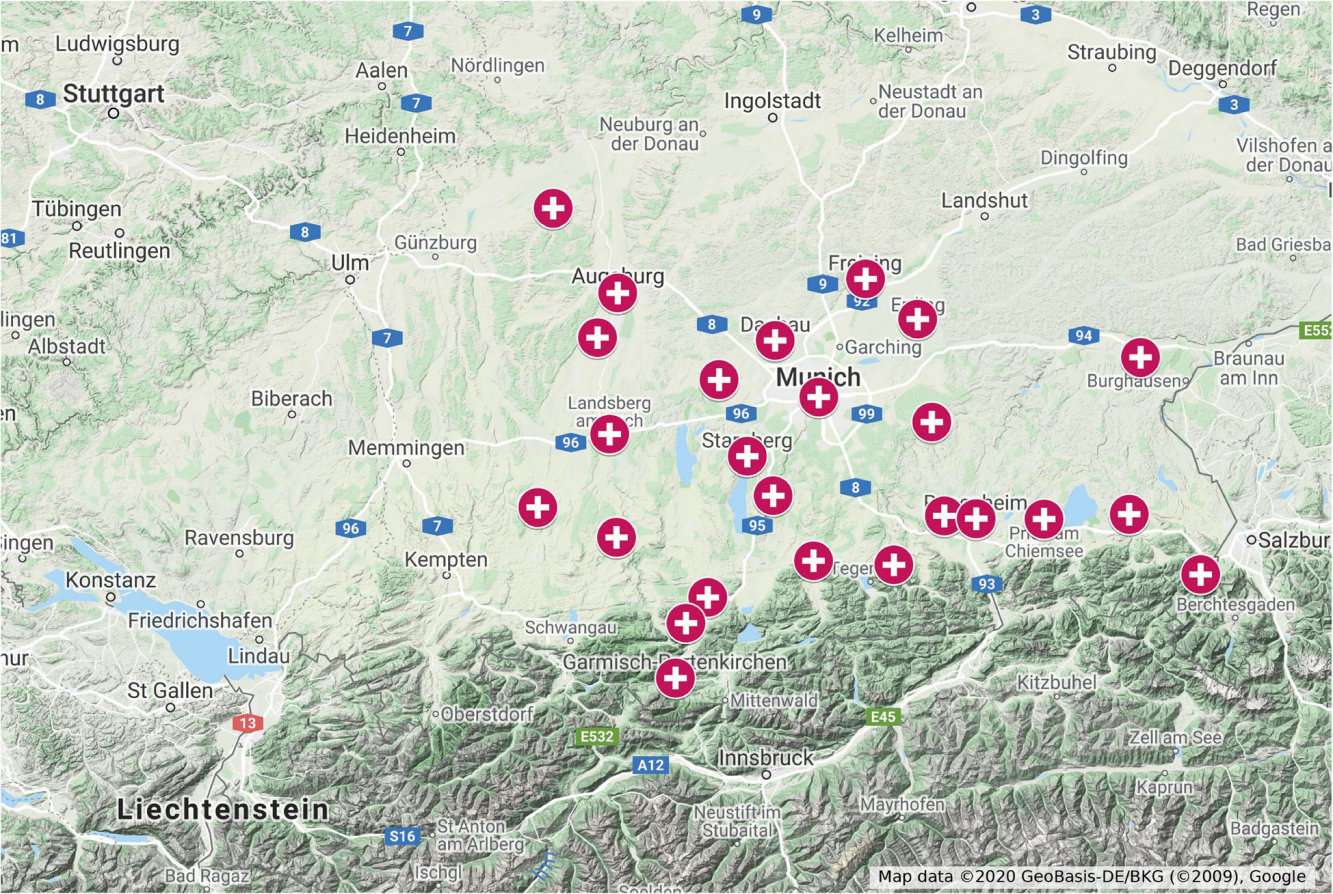
Catchment area of the participating hospitals in Southern Bavaria [19]. Cities of the 37 included hospitals: Hausham, Bad Tölz, Murnau, Munich (12 hospitals), Dachau, Schongau, Weilheim, Augsburg, Freising, Fürstenfeldbruck, Garmisch-Partenkirchen, Erding, Landsberg am Lech, Starnberg, Traunstein, Altötting, Bad Reichenhall, Ebersberg, Wertingen, Wolfratshausen, Kaufbeuren, Bad Aibling, Prien, Rosenheim, Bobingen. This figure was created with Google My Maps, Kartendaten © 2020 GeoBasis-DE/BKG (©2009)

In this study, we included patients with an Injury Severity Score (ISS) of at least 9 with post-ER admission to ICU or prior to death in the ER from 2013 to 2016. The patients were treated in 37 hospitals in Southern Bavaria as a typical foehn wind region (Fig. 2).

### Meteorological data

All meteorological data were obtained from a subunit of the German Meteorological service, Research Centre Human Biometeorology in Freiburg (DWD – Deutscher Wetterdienst).

For the presence of a foehn event in Southern Bavaria, the air pressure distribution in Europe has to generate a circulation directed from south to north over the Alps. Because there is no direct registration of foehn winds in Germany, a model had to be calculated from the standardized measured data which approximates the characteristics of foehn-typical circulation. The objective meteorological parameters were air pressure north and south of the Alps, wind direction and wind velocity measured on a determined summit of the Alps.

In detail, three criteria have to be met for the presence of foehn winds:

1. The difference in air pressures between weather stations south (Bolzano and Trento, Italy) and north (Munich, Germany) of the Alps was calculated. For a foehn event, a difference of 6 hPa between south and north is needed.
2. Wind direction and wind velocity were measured at the weather station on the summit of the Zugspitze. A wind directed from south to north (between 110° and 250°) and a mean wind velocity of at least 12 m/s are required for the generation of a foehn situation.
3. For defining a day as characterized by a foehn circulation, both criteria have to be present for at least six hours.

### Analysis

Patients, who were admitted via resuscitation room to one of the 37 hospitals and fulfilled the inclusion criteria of the TraumaRegister DGU**®** on a day with foehn-typical circulation were identified and characterized by demographic data like sex, age and ISS. In a next step, we calculated the influence on hospital admission of the season, day of the week and foehn circulation. A multivariate regression analysis was performed.

Statistical testing was performed using SPSS Software by IBM (New York, USA). The level of significance was set at *p =* < 0.05.

## Results

Between 2013 and 2016, 6215 individuals from 37 trauma hospitals in Bavaria met the inclusion criteria and were enrolled in this study. On 1461 days in total (4 years), a foehn-typical circulation was present on 65 days (4.5%). On the remaining 1396 days (95.5%), the meteorological data was not suitable for the presence of foehn winds.

**Fig. 3:**
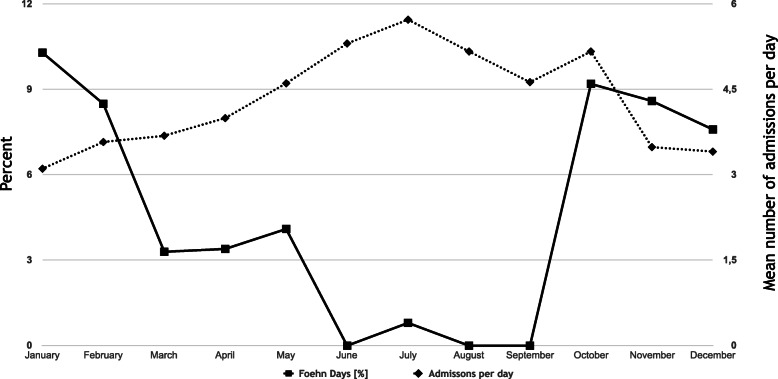
Average daily admissions per month and presence of foehn days per month in percent

Figure 3 illustrates the presence of foehn wind in dependence of each month. The data indicate that the main proportion of foehn days occurred in fall and winter months. Among the 6215 individuals, 301 patients (4.8%) suffered a severe trauma on foehn days. Of those, 65.1% were male and 34.9% were female. On days without foehn, 70.2% were of male and 29.8% of female gender. There was no significant difference between foehn days and no foehn days (*p =* 0.063) according to gender. The mean ISS was 20.2 (9–75) on foehn days vs. 20.3 (9–75) on days without foehn circulation (*p* = 0.99). On foehn days, 63.8% were admitted with an ISS of at least 16. On non-foehn days, 61.4% of the patients presented with an ISS of ≥16 (*p* = 0.41). The main proportion of the study population was admitted due to road traffic accidents (49.8% on foehn days, 51.5% on non-foehn days; *p* = 0.61).

The number of suspected suicides was almost equal in both groups (5.3% on foehn days vs. 4.4% on days without foehn-typical circulation; *p* = 0.47). For more details, please see Table 1.

**Table 1:** Study population

	Foehn	No foehn	Total	***p*** value
**Admitted patients**	301 (4.8%)	5914 (95.2%)	6215 (100%)	–
**Mean admitted patients per day**	4.6	4.2	4.3	0.57
**Days**	65 (4.5%)	1396 (95.5%)	1461 (100%)	–
**Age**	55 (6–96)	52 (0–99)	52 (0–99)	0.027
**Children (age 0–15)**	7 (2.3%)	130 (2.2%)	137 (2.2%)	0.88
**Male**	196 (65.1%)	4149 (70.2%)	4345 (69.9%)	0.063
**ISS**	20.2 (9–75)	20.3 (9–75)	20.3 (9–75)	0.99
**ISS ≥ 16**	192 (63.8%)	3633 (61.4%)	3825 (61.5%)	0.41
**Traffic-related injuries**	134 (49.8%)	1922 (51.4%)	2056 (51.3%)	0.61
**Penetrating injuries**	10 (3.6%)	174 (4.6%)	184 (4.5%)	0.43
**Severe head trauma**	124 (41.1%)	2499 (42.3%)	2623 (42.3%)	0.72
**Suspected suicides**	15 (5.3%)	169 (4.4%)	184 (4.5%)	0.47

A detailed analysis of the injury mechanisms in fall and winter months (main proportion of foehn days over the year, Fig. 3) is illustrated in Table 2. However, there was no significant (*p* = 0.33) difference regarding the injury mechanism in dependence of a foehn typical circulation.

**Table 2:** Injury mechanisms in fall and winter months in relation to no foehn days and foehn days. *P* = 0.33

	Foehn	No foehn	Total
**Car accidents**	57 (19.9%)	834 (21.4%)	891 (21.3%)
**Motorcycle accidents**	24 (8.4%)	405 (10.4%)	429 (10.3%)
**Bicycle**	25 (8.7%)	363 (9.3%)	388 (9.3%)
**Pedestrian**	18 (6.3%)	253 (6.5%)	271 (6.5%)
**Falls > 3 m**	48 (16.8%)	702 (18.0%)	750 (18.0%)
**Falls < 3 m**	72 (25.2%)	937 (24.1%)	1009 (24.2%)
**Others**	42 (14.7%)	397 (10.2%)	439 (10.5%)
**Total**	**286 (100%)**	**3891 (100%)**	**4177 (100%)**

On average, 4.3 patients suffered a severe trauma per day. On foehn days, the mean number of admitted patients was 4.6. On days without a foehn-typical circulation, the mean number of daily admitted patients was 4.2.

Focusing on daily trauma admissions without dependence of foehn winds, on average, 5.4 patients were admitted in summer (368 days), whereas 3.4 individuals were admitted in the winter months (361 days). 5.7 patients treated in July constituted the maximum vs. 3.1 in January as minimum number. For details please see Table 3. Figure 3 depicts the average number of daily admitted patients per month and the presence of foehn wind.

**Table 3:** Mean daily severe trauma admissions per month without dependence of the presence of foehn winds

Month	Average number of daily admitted patients	Standard deviation
January	3.1	2.0
February	3.6	2.3
March	3.7	2.2
April	4.0	2.0
May	4.6	2.7
June	5.3	2.8
July	5.7	2.4
August	5.2	2.6
September	4.6	2.3
October	4.3	2.7
November	3.5	2.1
December	3.4	2.0
Total	4.3	2.5

The incidence of severe trauma was highest on Saturdays (4.8 patients). On Tuesdays, the number of daily admitted patients was the lowest (3.9 patients). For further details, please see Table 4.

**Table 4:** Mean daily severe trauma admissions per weekday

Day of the week	Average number of daily admitted patients	Standard deviation
Monday	4.3	2.2
Tuesday	3.9	2.4
Wednesday	4.0	2.5
Thursday	4.3	2.5
Friday	4.4	2.4
Saturday	4.8	2.7
Sunday	4.1	2.6
Total	4.3	2.5

Performing a multivariate regression analysis with the number of daily patients admitted being the primary target value in dependence of spring, summer, fall, Saturdays and presence of foehn winds revealed a significant influence of a foehn-typical circulation on suffering a severe trauma (*p* = 0.004). The impact of spring (*p =* < 0.001), summer (*p =* < 0.001), fall (*p* < 0.001) and Saturdays (*p* = < 0.001) was significant as well. The results show a daily increase of 1.07 admitted patients in spring (95% confidence interval 0.72–1.42), 1.98 (95% confidence interval 1.63–2.32) in summer, 0.63 in fall (95% confidence interval 0.28–0.97) and 0.59 admitted patients on Saturdays (95% confidence interval 0.24–0.93). On days with foehn-typical circulation, an additional 0.87 individuals (95% confidence interval 0.23–1.47) suffered a severe trauma. For details please see Table 5.

**Table 5:** Multivariate linear regression analysis based on all 1461 days (2013–2016), with number of severe trauma admissions per day as dependent variable. Winter season serves as reference (constant term)

	Regression coefficient	Standard error	***p*** value	95% confidence interval
**Constant**	3.21	0.13	<.001	2.95–3.46
**Spring**	1.07	0.18	<.001	0.72–1.42
**Summer**	1.98	0.18	<.001	1.63–2.32
**Fall**	0.63	0.18	<.001	0.28–0.97
**Saturday**	0.59	0.18	0.001	0.24–0.93
**Foehn**	0.87	0.30	0.004	0.23–1.47

A more complex multivariate regression analysis additionally including every weekday (Monday-Sunday) and every month (January–December) as dependent variables revealed a number of 0.72 (*p* = 0.015; 95% confidence interval 0.13–1.3) severe trauma admissions on foehn days.

## Discussion

This study focuses on the influence of the external factor foehn wind when suffering a severe trauma defined as ISS ≥ 9.

For the first time, we were able to demonstrate that the presence of a foehn-typical circulation potentially increases trauma-induced hospital admission. Further, we determined the impact of seasons and the influence of the day of the week. In summer, spring and on Saturdays, the trauma-induced hospital admission is increased, whereas during fall and winter months, the daily number of admitted patients decreases.

Many studies investigated the influence of adverse weather events on the workload of trauma treatment facilities with different levels of impact of the respective conditions. Heat and high temperature are associated with an increase of hospital admissions due to trauma [20]. Especially pediatric subgroups appear to be more sensitive to a rise in temperature compared to adults. High temperature leads to an increase of 1.8% of adult trauma admission [15]. The increase of pediatric admission even accounts for up to 11% [21]. However, we were not able to identify a significant effect of a foehn typical circulation on pediatric trauma admissions. With higher temperatures during summer, trauma load is expected to be higher. According to Pape-Köhler et al., 39% of all trauma cases are registered during summer. In July, the trauma load is the highest at 10% [22]. They also observed a 2-fold increased admission for trauma in summer compared to winter. In this study, the results were quite similar. The daily admitted number of patients was the highest in July (5.7) and the lowest in January (3.1). Bhattarcharyya et al. also stated July and August to be the months with an anticipated high trauma volume independent of high temperature [23]. Furthermore, we discovered the opposite influence of winter with less admissions during the cold months (Table 3). This effect is obviously caused by an increased activity level in months with good weather conditions. Bad weather conditions in winter do not seem to influence the overall daily trauma load. The data used by Pape-Köhler et al. derived from a different time period from the same register which was used for this study. However, studies from the United States and Norway present similar results with an increased trauma incidence in summer [24, 25].

Focusing on the day of the week as an external factor for trauma-induced hospital admission, we discovered a minor peak on Saturdays compared to other weekdays. Tuesday was the day with the lowest number of injury-related hospital admissions (on average 3.9 daily admitted patients). In line with other studies, we expect increased free time activity and so called disco journeys on Saturdays to be responsible for these results [22, 26]. A younger patient age and an increased hospital admission during the night substantiate this theory [27, 28].

The general influence of a nature phenomenon on health conditions might be considered critically as people tend to correlate many different symptoms with the presence of weather events. However, these theories that are often perceived to be ludicrous are yet subjects of seriously conducted studies. For example, the influence of different lunar phases is well-investigated. Studies revealed higher aggressiveness, less sleep, increased crime rate and higher numbers of homicides during full moon [29–31]. However, an increased incidence of trauma was refuted [22, 32, 33]. The better illumination of highways and brighter nights might lead to less accidents. Thus, we excluded lunar phases from our regression analysis. Regarding the influence of wind on trauma, the amount of publications is limited. Schieman et al. correlated the incidence of spontaneous pneumothoraxes with the presence of foehn-similar Chinnok winds in Calgary [34]. There was no significant correlation. Yeung et al. identified an increased fall-injury volume during post-Chinook weather potentially caused by a higher prevalence of night freezing events [35].

O’connor et al. investigated the effect of wind speed on trauma. They were not able to detect any connection between wind speed, ISS, trauma admissions, Glasgow Coma Scale and length of hospital stay [36]. However, Parsons et al. demonstrated that an increase in wind speed results in a decrease of pediatric trauma patients [15].

This study is the first to investigate the influence of foehn winds on the possibility of suffering a severe trauma.

Conducting a multivariate regression analysis, we were able to identify an independent impact of a foehn-typical circulation on daily trauma admissions. In our study collective there was a significant increase of hospital admissions of trauma patients who presented an ISS ≥ 9. The effect of foehn winds on hospital admissions was similar to the impact of Saturdays, spring, summer and fall. The effect of foehn winds on health is described in several studies. However, the results are divergent. Authors describe that foehn winds lead to general illness symptoms (headache, tiredness, insomnia, nausea, circulatory dysfunctions). For example, Maciejczak et al. discovered an increased volume of admitted patients to cardiology wards with no impact on the incidence of actual cardiac events on foehn days [37]. However, Posse et al. were able to exclude an effect of foehn days on the number of notifications of illness in the Munich population over a period of 3 months in the 1970s [38]. Hence, it is difficult to blame the potential physiological effect of foehn winds for the observations of this study.

We rather expect the good visibility during a foehn-typical circulation with higher temperatures to be responsible for increased free time activity, thus resulting in a higher incidence of free time-associated accidents. With use of our meteorological model the majority of foehn days occurred in fall and winter months. This might lead to additional increased free time activity in the otherwise so called dark months. There was no significant influence of foehn winds on the severity (ISS) and cause of injuries. The TraumaRegister DGU**®** aims to include severely injured patients, which might explain the slightly higher percentage of patients presenting with an ISS ≥ 16 in both groups. Minor injuries are not recorded by the TraumaRegister DGU**®**, so we are not able to detect any influence of foehn winds on this large collective.

This study is not without limitations. Foehn winds are not directly measured by German weather stations. Thus, there is no reliable data to determine definitive foehn events over the course of several days. However, the model developed by the German Meteorological Service and used in this study represents a suitable analysis for the registration of a foehn-typical circulation, which is the condition for foehn winds in the valley. Due to the coverage of only one large geographic area (north to south: 120 km, west to east: 170 km), it is not possible to guarantee that foehn winds were present on the respective foehn days in every region of the 37 hospitals. For future studies it would be interesting to conduct a complete meteorological analysis which merges subjects and results of existing studies such as extreme wind velocities, foehn winds, precipitation, high and low temperatures or freezing events.

As additional limitation, local factors such as festivals and holidays could cause potential bias as they differ in the respective regions. As an additional limitation, this study used a register for data analysis. Hence we cannot guarantee for the data delivered by the participating hospitals. However, the TraumaRegister DGU**®** conducts audits to ensure high quality standards. Not all hospitals are required to deliver data to the TraumaRegister DGU**®**, which could cause a potential selection bias.

## Conclusion

In summary, we were able to confirm more trauma-related hospital admissions on days with more free time activities like Saturdays and in summer and spring. Furthermore, we demonstrated the influence of foehn winds on health related issues. We detected an increased hospital admission on days with a foehn-typical circulation. On a mean of 16 foehn days per year, 14 patients suffer a trauma in association with foehn wind, independent from the season and the day of the week. This impact appears to be minor but we advocate that hospitals should be prepared in terms of an optimized resource management to deal with a potentially increased trauma load comparable to periods with increased free time activities.

## Data Availability

The datasets used and analyzed during the current study are available from the corresponding author on reasonable request.

## Abbreviations

TR-DGU: TraumaRegister DGU®
DGU: Deutsche Gesellschaft für Unfallchirurgie
ER: Emergency room
ICU: Intensive care unit
AUC: Akademie der Unfallchirurgie GmbH
ISS: Injury Severity Score
DWD: Deutscher Wetterdienst
